# Application of Nanomaterials in the Deacidification of Paper-Based Cultural Heritage

**DOI:** 10.3390/nano16040221

**Published:** 2026-02-07

**Authors:** Chun Kong, Jinxiu Song, Yu Tong, Tao Chen, Sheng Chen

**Affiliations:** 1 Centre for the Protection of Cultural Property, Ningbo University of Finance & Economics, Ningbo 315175, China; kongchun@nbufe.edu.cn (C.K.); tongyu@nbufe.edu.cn (Y.T.); 2 Institute of Tribology, Hefei University of Technology, Hefei 230009, China

**Keywords:** nanomaterials, paper-based cultural heritage, deacidification, cultural heritage conservation, alkaline reserve

## Abstract

Acidity is a primary factor leading to the deterioration of paper-based cultural heritage, and deacidification treatment is a crucial preventive conservation measure for extending their lifespan. Traditional deacidification techniques, such as the particle suspension method and vapor phase method, have limitations in terms of penetration uniformity, treatment efficacy, or safety. Nanoscale alkaline materials, represented by nano-calcium hydroxide and nano-magnesium hydroxide, offer an innovative solution with the potential to achieve more uniform, efficient, and long-lasting paper deacidification, owing to their high specific surface area, enhanced reactivity, and superior penetration capacity derived from the nanoscale dimension. It is important to note that the realized uniformity and depth of treatment are contingent upon substrate properties (e.g., fiber density, porosity) and application parameters. This paper provides a systematic review of the main types of nanomaterials applied in the deacidification of paper artifacts—including their synthesis and dispersion stabilization methods—application techniques (such as immersion and spraying) and performance evaluation systems (including pH value, alkaline reserve, and mechanical properties). Through comparative analysis and case studies, the advantages and current challenges of nano-deacidification technology are elaborated. Finally, future directions for nano-deacidification technology are discussed, particularly focusing on material optimization, standardized evaluation, and prospects for scalable application tailored to the practical needs of cultural heritage conservation.

## 1. Introduction

Paper-based cultural heritage, including ancient books, documents, calligraphy, paintings, and archives, serves as a fundamental carrier of human civilization and historical memory, possessing significant historical and artistic value [1,2]. Paper, as its substrate material, is primarily composed of cellulose—a natural polymer that is highly susceptible to hydrolysis under acidic conditions. This reaction leads to the cleavage of molecular chains and a decrease in the degree of polymerization, resulting in loss of paper strength, yellowing, embrittlement, and eventual disintegration [3,4]. The acidification of paper stems from two main categories of causes. The internal factors include sulfuric acid generated from the hydrolysis of residual alum in modern papermaking processes [5,6] and organic acids produced by the oxidation of lignin present in the raw materials [7,8]. External factors involve improper storage conditions—such as fluctuations in temperature and humidity, exposure to light, and microbial metabolism—as well as contamination from acidic adhesives used in earlier restoration practices [9,10,11]. Furthermore, the structure and degradation state of paper are often non-uniform. Factors such as surface sizing treatments and gradients in degradation (e.g., increased acidity at edges or in foxing spots) create variability in porosity, permeability, and chemical reactivity across an artifact. Therefore, deacidification has become a critical procedure in the preventive conservation of paper-based artifacts. Its objective extends beyond merely neutralizing free acids present in the paper; it aims to establish an alkaline reserve within the material to continuously counteract potential acid formation in the future. This mechanism effectively inhibits the hydrolytic degradation of cellulose over the long term, thereby significantly extending the lifespan of cultural heritage items [12].

Traditional deacidification processes primarily include liquid-phase and gas-phase methods. In liquid-phase methods, early treatments using calcium hydroxide-calcium bicarbonate aqueous solutions often led to paper wetting, ink feathering, and cockling or wrinkling after drying [13,14]. Later developed organic solvent systems, such as the magnesium-titanium isopropanol composite system (known as the Wei T’o method), improved wetting issues but were associated with solvent toxicity and residual odor problems [15,16,17]. Gas-phase methods, exemplified by diethyl zinc treatment, offered excellent permeability but were limited by high equipment costs, operational hazards, and strong corrosiveness, and are now rarely used [18,19]. Currently, the widely adopted approach is the particle suspension method, which involves dispersing micrometer-sized alkaline particles (e.g., magnesium carbonate, magnesium oxide) in inert liquids (such as perfluoroheptane) to form a suspension for paper treatment. While this method shows significant effectiveness, the limited penetration depth of micrometer-sized particles, uneven dispersion, and tendency to accumulate on the paper surface make it difficult to achieve uniform deacidification throughout bound books [20,21].

The advancement of nanomaterials science has provided novel and effective solutions for the deacidification of paper-based cultural relics. When the size of alkaline deacidifying agents is reduced to the nanoscale, their specific surface area increases by orders of magnitude, accompanied by a significant enhancement of surface activity. Moreover, nanoparticles can penetrate the fiber network of paper more effectively than their micrometer-sized counterparts, potentially addressing the uniformity challenges associated with traditional agents [22,23]. This improved penetration is facilitated by their reduced size, but its efficacy is contingent upon a complex interplay of factors including nanoparticle surface chemistry, the properties of the dispersion medium (e.g., surface tension, viscosity), and the specific porosity, sizing, and degradation state of the historical paper substrate. Additionally, nanomaterial-based deacidification technology has evolved from a single deacidification function toward the synergistic development of multifunctionality, integrating deacidification, reinforcement, and antibacterial properties [24,25,26]. It should be noted, however, that while such multifunctional systems have been successfully demonstrated in laboratory settings, their integration into standardized conservation practice—especially for complex, heterogeneous heritage objects—requires further validation and adaptation to real-world conditions. Researchers have optimized the performance of nanomaterial deacidification agents through various preparation techniques. For instance, Giorgi et al. developed magnesium hydroxide nanoparticles via a homogeneous precipitation method. By adjusting the type of magnesium salt counterions, reaction temperature (25–90 °C), and salt concentration (0.2–1 M), they achieved precise control over particle size (50–200 nm) and successfully formulated a stable dispersion in 2-propanol [27]. Poggi et al. employed a solvothermal reaction using metallic calcium and short-chain alcohols (ethanol, n-propanol) to prepare high-concentration, highly crystalline calcium hydroxide nanoparticle dispersions (with average particle sizes of approximately 80 nm + 220 nm in the E system and around 260 nm in the 1P system), which could be directly applied without additional purification [28]. Chen et al. developed a hybrid fluorocarbon solvent system comprising perfluorobutyl methyl ether (PFM) and perfluorohexanone (PFH). By adding 0.5 wt% surfactant and applying ultrasonic dispersion (20 kHz, 15 min), they obtained a stable suspension of nano-MgO (30 nm) for deacidification, effectively inhibiting particle agglomeration [12]. These materials not only precisely adjust the pH of paper to the safe range of 7.8–8.2 but also simultaneously improve its mechanical properties. For example, after treatment with magnesium hydroxide nanoparticles, hydrothermally aged paper exhibited a fracture bond percentage as low as 0.33%, with almost no loss in tensile strength (ΔTS ≈ −0.01%, the estimated measurement error for TS is about 5%) [27]. Calcium hydroxide nanoparticles stabilized the pH of acidified paper and canvas at 6.8–7.2, raised the pyrolysis temperature by approximately 20 °C (the slight difference between samples is within the experimental error of ±1 °C), and significantly enhanced thermal stability [28]. Papers treated with the hybrid fluorocarbon solvent system showed an alkaline reserve of up to 0.709 mol/kg. After dry-thermal aging, all performance indicators declined by less than 17%, and color difference (ΔE) was controlled within an acceptable range of 1.8–2.1, meeting conservation requirements [12]. These process-optimized nanomaterials can be precisely tailored to meet the specific conservation needs of different types of paper-based cultural relics, such as mechanical pulp paper and handmade paper. For example, dispersions of trimethylsilyl cellulose-stabilized magnesium hydroxide nanoparticles in hexamethyldisiloxane are used for historical wood pulp paper [29]. And nano-calcium hydroxide or nano-calcium carbonate in alcohols are better for modern lignocellulosic paper [30]. The choice is tailored to the paper’s fiber type, sizing, initial pH, and the sensitivity of its media. Nanomaterial-based deacidification (using nanoparticles such as nano-calcium hydroxide and magnesium hydroxide) has emerged as a cutting-edge research focus in the field of cultural heritage conservation, with continuous progress being made in material preparation optimization, performance evaluation, and practical applications.

**Figure 1:**
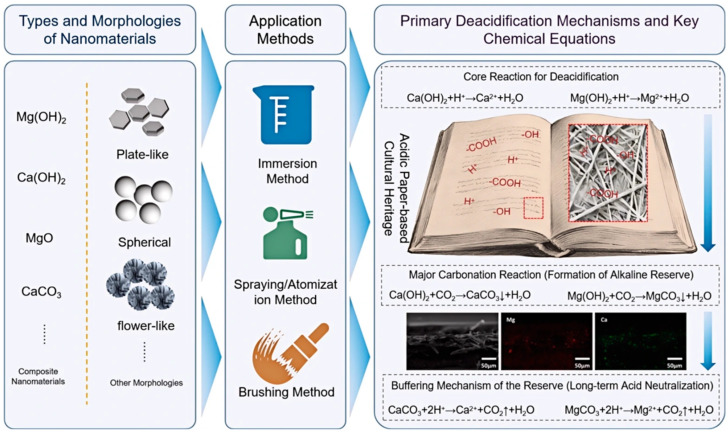
Schematic illustration of primary material types, application methods, and deacidification principles for paper artifacts.

This paper aims to systematically review the research and application progress of nanomaterials in the deacidification of paper-based cultural heritage (Figure 1), with a focus on evaluating the types and preparation techniques of nano-deacidification materials, as well as their application methods (e.g., immersion, spraying). The analysis is centered on key performance indicators such as pH value, alkaline reserve, mechanical strength, color difference, and aging resistance. Furthermore, this review places a stronger emphasis on elucidating the underlying mechanisms of nanoparticle-paper interactions, highlighting recent trends toward multifunctional nanocomposites, and critically discussing the practical translation challenges from laboratory to conservation practice. Finally, current technical challenges and future development directions are discussed, aiming to provide comprehensive and precise reference for both heritage conservation professionals and materials researchers.

## 2. Types, Preparation, and Characteristics of Nanoparticle-Based Deacidification Agents

Nanomaterials applied to paper deacidification are essentially nanoparticles with high alkalinity and high reactivity. The core requirements include high purity, nanoscale size, good dispersion stability, excellent compatibility with paper fibers, and non-toxicity ensuring the safety of cultural heritage artifacts.

### 2.1. Nano-Calcium Hydroxide

**Figure 2:**
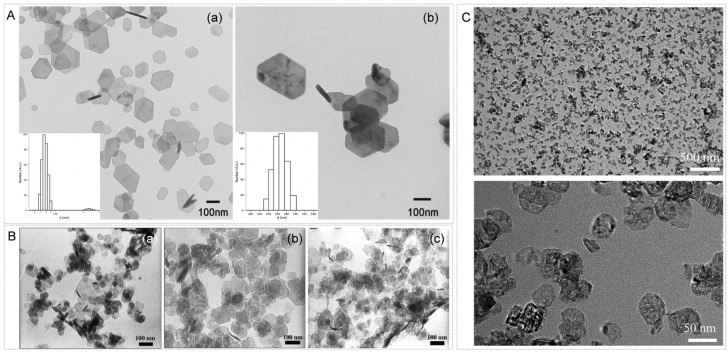
(**A**) Transmission electron microscopy (TEM) image and size distribution (**a**) of Ca(OH)_2_ nanoparticles dispersed in ethanol. TEM image and size distribution (**b**) of Ca(OH)_2_ nanoparticles dispersed in isopropanol [28]. (**B**) TEM images of Mg(OH)_2_ nanocrystals synthesized in aqueous solution at 90 °C using magnesium nitrate (**a**), magnesium chloride (**b**), and magnesium sulfate (**c**) as source solutions [27]. (**C**) TEM micrograph of the MgO/CaCO_3_ nanocomposite (with an MgO to CaCO_3_ ratio of 3:1) [40].

Nano-calcium hydroxide is the most extensively studied and widely used nanomaterial for deacidification (Figure 2A). Its deacidification mechanism is well-defined: first, Ca(OH)_2_ nanoparticles neutralize free acids in the paper; subsequently, during post-treatment storage, the residual Ca(OH)_2_ slowly reacts with carbon dioxide in the air to form nano-calcium carbonate, which deposits onto the paper fibers [28,31,32].

Ca(OH)_2_ + H^+^ → Ca^2+^ + H_2_O

Ca(OH)_2_ + CO_2_ → CaCO_3_ + H_2_O

As a mild alkaline buffer, the generated CaCO_3_ can effectively and sustainably counteract future acidification threats [28,32]. It is important to note that carbonation proceeds partially during the application and drying stages but continues slowly over years within the paper matrix, thereby contributing to a long-term alkaline reserve.

#### 2.1.1. Preparation Methods

Chemical Precipitation Method: This is the most commonly used approach. Water-soluble calcium salts such as calcium chloride or calcium nitrate are reacted with sodium hydroxide as precursors at temperatures ranging from room temperature to 90 °C. By controlling parameters such as supersaturation and stirring speed, calcium hydroxide precipitate is formed. After washing and drying, the product is obtained. Hexagonal plate-like crystals with sizes of 50–350 nm can be prepared without surfactants. For smaller particle sizes (30–60 nm), surfactants such as CTAB can be added, or microemulsion methods may be employed. This method is suitable for applications such as cultural heritage conservation and material reinforcement [33,34].

Solvent Exchange Method: A preparation strategy more suitable for cultural heritage conservation applications. Calcium hydroxide nanoparticles are first synthesized in an aqueous phase via chemical precipitation, followed by centrifugal washing. The water phase is then gradually replaced with short-chain alcohols such as isopropanol or ethanol. The alcohol medium, with its low surface tension and good wettability, can penetrate paper pores effectively and evaporates quickly. It is compatible with cellulose and traditional inks, avoiding the formation of white efflorescence caused by particle settling. Ultimately, a stable alcohol-phase suspension is obtained [28,33,35].

#### 2.1.2. Advantages and Challenges

Advantages: Strong alkalinity (pH~12.4) with high acid-neutralization capacity; the final product CaCO_3_ is chemically highly stable, providing long-lasting alkaline reserve; relatively low cost [28,35].

Main Challenges: Ca(OH)_2_ readily reacts with CO_2_ in air, potentially converting prematurely to calcium carbonate during storage and application, thereby reducing its reactivity. Consequently, the long-term stability of nano-Ca(OH)_2_ suspensions remains a key research and practical challenge. Typically, sealed storage at low temperatures or exploration of more stable dispersion systems is required [33,35]. Instability during storage can lead to particle aggregation, which may compromise penetration uniformity and result in inconsistent alkaline reserve deposition, thus affecting treatment reproducibility.

### 2.2. Nano-Magnesium Hydroxide

Nanoscale magnesium hydroxide serves as an important alternative to nanoscale calcium hydroxide (Figure 2B). Its deacidification mechanism is similar, ultimately converting to magnesium carbonate to provide buffering capacity [36].

Mg(OH)_2_ + 2H^+^ → Mg^2+^ + 2H_2_O

Mg(OH)_2_ + CO_2_ → MgCO_3_ + H_2_O

#### 2.2.1. Preparation Method

Regarding preparation, the chemical precipitation method is commonly employed, where soluble magnesium salts (e.g., magnesium chloride, magnesium sulfate) react with alkali to produce the desired material. Through process control, various nanostructures such as nanosheets, nanoflowers, or nanospheres can be obtained [27,37,38].

#### 2.2.2. Advantages and Challenges

Advantages: Magnesium hydroxide exhibits a milder alkalinity (pH~10.5) compared to Ca(OH)_2_, potentially causing less damage to paper fibers and certain sensitive pigments. Both Mg(OH)_2_ and MgCO_3_ have lower molar masses, meaning a greater alkaline equivalent per unit mass. Some studies have also reported better dispersion stability for nanoscale Mg(OH)_2_ [27,39].

Challenges: Magnesium carbonate is less stable than calcium carbonate. Under humid conditions, it may partially convert to more soluble basic magnesium carbonates. Therefore, the long-term stability of its buffering behavior requires more in-depth investigation [39]. In particular, under fluctuating relative humidity, magnesium carbonate can undergo hydration–dehydration cycles that may alter its crystallinity and buffering capacity, posing a potential trade-off against its milder initial alkalinity.

To facilitate the selection of appropriate nano-alkaline materials for specific conservation scenarios, Table 1 provides a direct comparison between nano-Ca(OH)_2_ and nano-Mg(OH)_2_ across several key dimensions.

In practice, the choice between the two often depends on the artifact’s condition, medium sensitivity, and desired balance between immediate neutralization strength and long-term buffer stability. Hybrid or composite systems are increasingly explored to combine their respective advantages.

### 2.3. Other Nano-Alkaline Materials

Nano-magnesium oxide: Possesses a higher alkali reserve capacity and can serve as an alkaline reserve material. Its hydration process can slowly generate Mg(OH)_2_, although the reaction rate is relatively slow [42,43,44].

Nano-calcium carbonate: It is the final form of alkaline reserve and can be directly introduced into paper as a buffering agent. However, it lacks initial strong neutralization capability and is typically used in combination with other methods or as a supplement [30,45].

Composite nanomaterials: To overcome the limitations of single materials, researchers have developed core–shell structures or composite materials [40,46]. For example (Figure 2C), MgO/CaCO_3_ nanocomposites, as novel paper deacidification agents, combine the immediate and efficient deacidification effect of MgO with the long-term, mild deacidification effect of CaCO_3_ [40].

### 2.4. Stability of Nano-Dispersion Systems

Regardless of the type of nano-alkaline material, it must maintain a uniform and stable suspension state within a dispersion medium prior to application to prevent particle agglomeration and sedimentation. Strategies for enhancing stability include: physical ultrasonication, where soft agglomerates are disrupted via ultrasonic treatment before use [47]; chemical modification, employing surfactants or coupling agents to functionalize the nanoparticle surface, thereby increasing steric hindrance or electrostatic repulsion [48]; solvent optimization, identifying dispersion medium systems that are more stable and environmentally friendly than isopropanol [49]; and nanofluid technology, which involves preparing highly concentrated, colloid-like stable nanoparticle suspensions and represents a recent research frontier [49]. Post-deposition aggregation within the paper matrix is possible, especially if the dispersion is unstable or if drying conditions promote particle migration. However, optimized dispersion systems and controlled application (e.g., multiple light sprays) can minimize such effects and promote uniform distribution.

## 3. Application Methods and Efficacy Evaluation of Nano-Deacidification Treatment

### 3.1. Application Technologies

Depending on the state of the artifact (single leaf, bound volume, or complete book) and its degree of fragility, different treatment methods can be selected.

#### 3.1.1. Immersion Method

**Figure 3:**
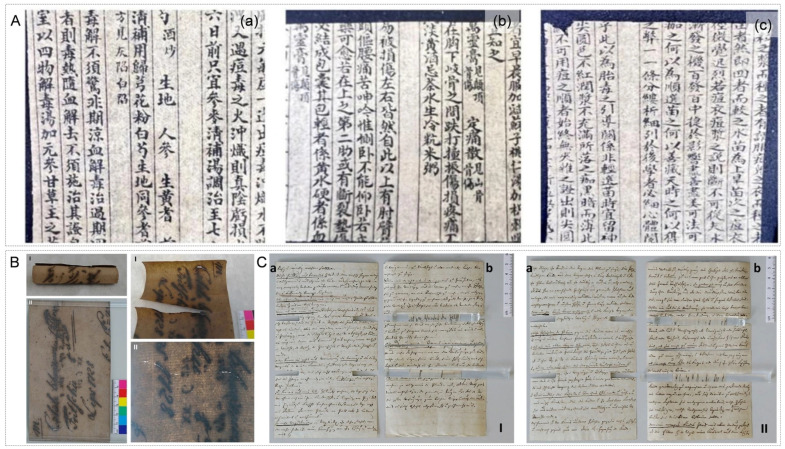
(**A**) Appearance of Qing Dynasty paper after deacidification with different material treatments: (**a**) magnesium oxide (MgO) deacidification group, (**b**) polyvinylpyrrolidone-immobilized magnesium oxide (MgO/PVP) deacidification group, (**c**) polyvinylpyrrolidone-modified magnesium oxide (MgO-PVP) deacidification group [53]. (**B**) Comparison of badly degraded paper (bad condition) before (**I**) and after (**II**) treatment. A 1% CNF B suspension was brushed onto both sides of the paper; the stabilizing effect of CNF at the fracture site is clearly visible [55]. (**C**) Treatment results of paper in fair condition (**a**: unaged sample, **b**: aged sample): (**I**) after chemical treatment (calcium phytate/calcium hydrogencarbonate baths) followed by brush-coating with 1% CNF B suspension as the final step; (**II**) brush-coating with 1% CNF B suspension prior to chemical treatment. The shadow on the right sample paper is caused by its curvature [55].

Single sheets of paper or separable documents are completely immersed in a nanoparticle dispersion for a period, then removed and dried. This method ensures the most thorough treatment with uniform nanoparticle penetration. However, it is only suitable for papers with sufficient strength to withstand liquid wetting and is not applicable to bound materials [50,51,52,53]. For sized papers or those with weakened fiber networks, immersion may cause fiber swelling, ink bleeding, or further mechanical damage. Therefore, pretreatment assessment is essential. Yao et al. developed a PVP-fixed nano-MgO material (MgO/PVP) and applied it via immersion in a fluorocarbon solvent (C_5_H_3_F_9_O) system for deacidifying paper from a Qing Dynasty medical text (Figure 3A). This treatment raised the paper pH to 8.85, increased tensile strength by 16.3%, and caused minimal color change [53]. While effective, the use of fluorocarbon solvents in such immersion processes requires careful risk assessment regarding operator exposure and environmental release, urging the development of greener solvent alternatives or closed-system application setups. In contrast to the aforementioned organic-phase dispersion systems, another line of research focuses on achieving efficient and stable dispersion of nano-alkaline materials in aqueous phases. Wang et al. synthesized ultrathin colloidal magnesium hydroxide nanosheets and applied an aqueous-phase immersion method to deacidify acidified bamboo paper, raising its pH from 5.03 to 7.29. Even after accelerated aging at 105 °C for five months, the paper maintained a pH of 5.47, demonstrating remarkable long-term anti-acidification performance [51].

#### 3.1.2. Spraying/Atomization Method

This technique employs an airbrush or specialized atomization equipment to apply the nanoparticle dispersion onto the paper surface in the form of a fine mist. It is suitable for fragile papers and some bound materials, as it reduces the amount of liquid applied. However, controlling penetration depth and uniformity is critical to avoid local overwetting [24,54]. Li et al. developed a bacterial cellulose/zinc oxide nanocomposite coating (BC/ZnO). Using the spraying method, they formed a uniform coating on the paper surface for deacidification, which also significantly enhanced the paper’s mechanical strength, anti-aging properties, and antifungal capabilities [24]. To address specific needs such as varying degrees of acidification, paper characteristics, and the protection of objects with water-sensitive inks, the atomization spraying method has also been utilized. Bicchieri et al. developed nano-calcium carbonate and nano-calcium propionate materials. For specific water-sensitive medieval papers, they employed atomization spraying (applying the material as an aqueous solution mist) for rapid deacidification. This method significantly increased the pH of the acidified paper and ink (by 3–5 units) without causing noticeable color changes, thereby achieving safe and effective protection for both the paper and the writing media [54].

#### 3.1.3. Brush Coating Method

A soft brush is used to gently apply the dispersion onto the paper surface. This method is simple but offers the poorest uniformity, easily leading to surface accumulation of particles. It is typically used only for local reinforcement treatments [55]. Poggi et al. developed a Mg(OH)_2_ nanoparticle deacidification system. This system utilizes alcohol-dispersed nanoparticles applied via brush coating to the paper surface, enabling precise pH adjustment in a non-aqueous medium. This effectively avoids the dissolution and bleeding of inks that can occur with aqueous treatments, providing a new strategy for the single-step combined conservation of paper artifacts containing iron gall ink [21]. This brush-coating-based precision application strategy has further inspired researchers to optimize treatment processes through composite systems. Völkel et al. developed an integrated method combining calcium phytate/calcium bicarbonate treatment with nanocellulose stabilization. They implemented a stepwise integrated process (incorporating nanocellulose by brushing or mixing it into the treatment solution within the phytate deacidification step) to achieve deacidification (Figure 3C). This approach effectively mitigated the chemical and mechanical degradation of paper caused by iron gall ink while preventing iron ion migration during treatment [55].

#### 3.1.4. Best Practices

For precious cultural relics, a common practice is the application of multiple light sprays. This ensures safety while gradually achieving the desired penetration and neutralization effect [56]. Wang et al. developed one-dimensional and two-dimensional weakly alkaline hydrated magnesium carbonate materials. They applied a multi-cycle dip-coating method for deacidification treatment (each paper sample underwent 5 immersion-drying cycles). This material system not only raised and maintained the pH of acidified paper within the safe range of 7.5–10 in the long term but also provided significant filling, reinforcement, and flame-retardant enhancement to the paper fibers. Additionally, it avoided the damage to alkali-sensitive pigments caused by traditional strong alkaline materials, achieving integrated deacidification, strengthening, and safe protection [56].

### 3.2. Efficacy Assessment Framework

A multidimensional assessment framework is essential for scientifically evaluating the efficacy of nanomaterial-based deacidification treatments for acidic paper artifacts. As summarized in Table 2, the core evaluation metrics and methodologies for nanomaterial deacidification performance are outlined below.

### 3.3. Mechanisms of Impact on Paper Structure and Properties

A successful nano-deacidification treatment is not merely a chemical process of acid-base neutralization but also a physicochemical process involving interfacial interactions.

#### 3.3.1. Acid Neutralization and Alkaline Reserve

Paper acidification stems from the accumulation of acidic groups such as carboxyl groups generated by cellulose hydrolysis. Nano-deacidifying agents (e.g., nanoparticles of MgO, CaO, Ca(OH)_2_, Mg(OH)_2_) can rapidly neutralize these acidic groups through acid–base reactions [41,65,66]. For instance, MgO reacts with the H^+^ from acidic groups to form soluble magnesium salts, which accomplishes acid elimination while avoiding excessive corrosion of fibers by strongly alkaline substances [67]. Simultaneously, unreacted nano-deacidifying agents and their neutralization products (e.g., MgCO_3_, CaCO_3_) remain within the fiber network, forming a long-lasting alkaline reserve. These stable inorganic mineral phases continuously capture acidic substances from environmental CO_2_ and SO_2_ dissolution, thereby maintaining the paper in a long-term, stable, slightly alkaline state (pH 7.5–9.0) [41,68]. For example, Cui et al.’s CaMgO_2_ composite deacidification system generates a CaCO_3_-MgCO_3_ composite mineral phase upon conversion. This system achieves rapid acid-base neutralization and maintains a sufficient alkaline reserve (0.153 mol/kg) after dry-heat aging. This effectively delays subsequent paper acidification and supports long-term preservation stability [65].

#### 3.3.2. Penetration and Deposition

**Figure 4:**
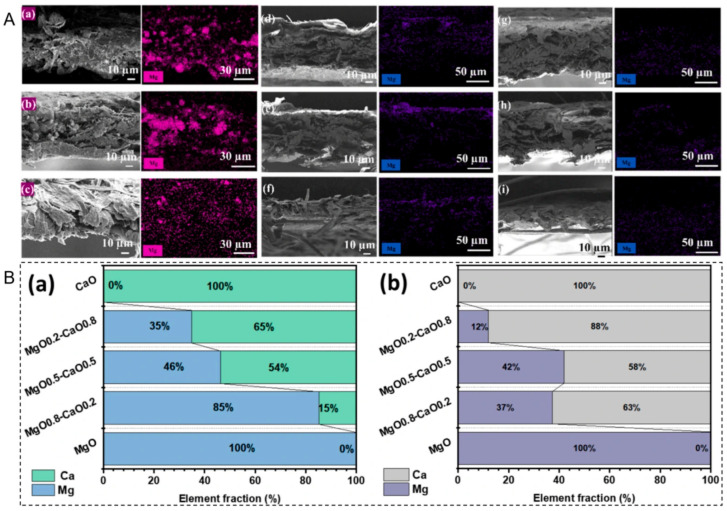
(**A**) Cross-sectional penetration of three types of MgO in paper after deacidification: SEM and EDS analysis of magnesium element distribution. ((**a**–**c**): Paper from the 1990s, Republican period, and Qing dynasty treated with F-MgO, respectively; (**d**–**f**): Paper treated with S-MgO; (**g**–**i**): Paper treated with FL-MgO) [57]. (**B**) Quantitative verification of interfacial binding effectiveness. Mass percentage of calcium and magnesium elements on the paper surface (**a**); Mass percentage of calcium and magnesium elements in the paper cross-section (**b**) [65].

Leveraging their small size, nanoparticles penetrate the paper fiber network via capillary action. They tend to deposit at fiber intersections and surface indentations. This deposition pattern provides a certain reinforcing effect on the bonding points between fibers [14,65]. Gui et al. developed a morphology-controlled nano-MgO paper deacidification system in fluorocarbon solvents. By preparing nano-deacidifiers with different morphologies such as flake-like (F-MgO), spherical (S-MgO), and flower-like (FL-MgO), they utilized morphological differences to optimize adsorption and penetration performance. Among them, flake-like MgO, due to its higher zeta potential, formed stronger electrostatic adsorption with the paper surface, leading to more uniform surface adsorption and superior cross-sectional penetration, effectively reducing agglomeration (Figure 4A). This provided key support for enhancing paper deacidification efficiency and long-term preservation stability [57].

#### 3.3.3. Interfacial Bonding

Nanoparticles such as Ca(OH)_2_, Mg(OH)_2_, and CaO primarily bind to cellulose fibers through physical adsorption and hydrogen bonding. After conversion to carbonates, the formed inorganic mineral phases create an organic–inorganic composite structure with the organic fibers. This structure can moderately enhance the rigidity of the paper without clogging its porous structure [65,68,69]. Cui et al. developed a CaMgO_2_ nanocomposite paper deacidification system based on an environmentally friendly fluorocarbon solvent (methyl perfluorobutyl ether). By adjusting the Mg/Ca molar ratio, they prepared a series of composite deacidifiers, with MgO_0_._2_-CaO_0_._8_ performing optimally (Figure 4B). The core of its interfacial bonding mechanism lies in the strong affinity of CaO for paper fibers, which promotes stable suspension of composite particles in the solvent (transmittance change < 5% over 9 h). The particles uniformly adhere to the fiber surface via physical adsorption and penetrate internally without reacting with the inherent functional groups of cellulose. After dry-heat aging, the system retained good alkalinity and mechanical strength. The treatment also showed minimal impact on ink (ΔE ≤ 1.16), aligning with the minimal intervention principle in cultural heritage conservation [65].

#### 3.3.4. Optical Impact

The size of nanoparticles is far smaller than the wavelength of visible light, so their inherent light scattering is limited. Unlike micron-sized particles, they do not significantly increase paper opacity or whiteness. Color changes mainly originate from trace impurities in the organic treatment solvents or minor structural changes in fibers induced during the treatment process [14,65,69]. He et al. developed a capillary-action-enhanced paper deacidification system based on nano-calcium carbonate (nano-CaCO_3_). By controlling its particle size to a narrow distribution of ~70 nm and optimizing solvent wettability, they strengthened the capillary penetration effect. Nano-CaCO_3_ itself exhibits excellent optical inertness and compatibility with paper. Its small particle size allows uniform penetration into fiber interstices via capillary action, avoiding localized agglomeration that could cause light scattering differences. Furthermore, the system does not introduce chemical components prone to causing discoloration, does not react with cellulose functional groups or ink pigments, and does not cause fiber swelling. The color difference (ΔE) of treated paper remains at an extremely low level. While achieving uniform deacidification and long-term anti-aging, the system maximally preserves the original whiteness and optical properties, conforming to the conservation principle of “repairing the old as the old” for paper cultural relics [14].

## 4. Case Studies and Analysis

### 4.1. Nano-Ca(OH)_2_ Treatment for Acidic Paper

Weng et al. developed a deacidification system for paper-based cultural relics based on subcritical 1,1,1,2-tetrafluoroethane (R134a) and Ca(OH)_2_ nanoparticles (particle size 60–90 nm, hexagonal platelet morphology), achieving integrated deacidification and cleaning. This system was applied to actual artifacts, including naturally aged mechanical wood pulp papers from the 1920s and 1940s (MWP-1920, MWP-1940) and a 1962 issue of Worker’s Daily (WDP-1962) containing ink and a red seal. It efficiently neutralized paper acidity, elevating both surface and internal pH to an optimal range (approximately 8.5). The resulting alkaline reserve (up to 1.213 mol/kg) was significantly higher than that achieved by traditional spraying methods. The color difference (ΔE) of treated artifacts was below 1.0 (imperceptible to the naked eye) with no ink bleeding observed. Furthermore, the treatment enhanced the tensile strength of the paper. After accelerated aging via dry heat (105 °C) and damp heat (80 °C), the treated samples maintained good pH stability and mechanical properties, effectively retarding cellulose degradation. This provides a mild, efficient, and long-lasting conservation solution for aged paper artifacts containing pigments [69].

**Figure 5:**
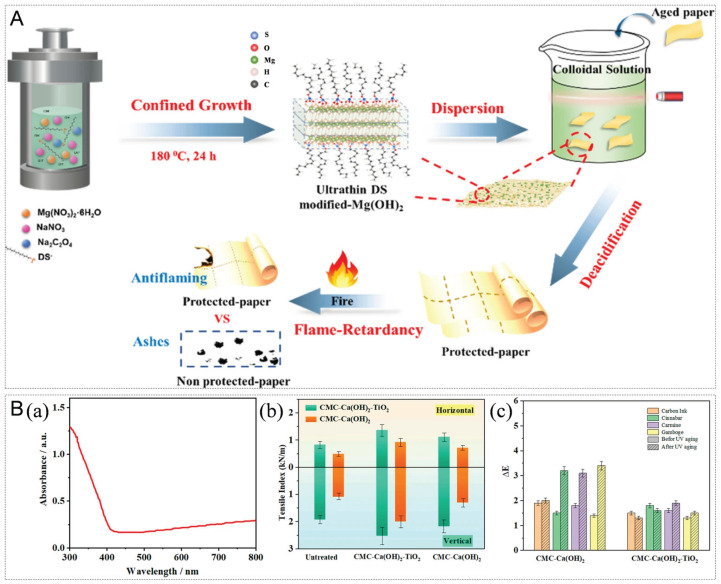
(**A**) Schematic illustration of the synthesis of magnesium hydroxide nanoflakes and their application for deacidification and flame-retardancy of paper-based relics [51]. (**B**) (**a**) UV-Vis diffuse reflectance spectroscopy of the CMC-Ca(OH)_2_-TiO_2_ composite material; (**b**) Changes in the cross-direction and machine-direction tensile indices of paper after UV aging, following treatment with CMC-Ca(OH)_2_ and CMC-Ca(OH)_2_-TiO_2_; (**c**) Color difference (ΔE*) changes of carbon ink, vermilion, magenta, and gamboge pigments before and after UV aging, following treatment with CMC-Ca(OH)_2_ and CMC-Ca(OH)_2_-TiO_2_ [50].

In the conservation of paper-based cultural relics, the application of nano-Mg(OH)_2_ aims to address acidification issues and explore additional protective functionalities. For instance, Meng et al. developed a GG-C-M nanocomposite hydrogel system. By integrating nano-Mg(OH)_2_ into a network of gellan gum and sodium carboxymethyl cellulose, this system not only provides a stable alkaline reserve (0.27–0.34 mol kg^−1^) for acidified paper (restoring pH from ≤5.0 to 7.25–7.32) but also significantly enhances its mechanical strength (tensile strength reaching 35.50–41.40 N), demonstrating a synergistic “deacidification-reinforcement” integrated strategy [46]. To achieve protection that is both durable and uniform, the nano-alkaline material must itself be stable in dispersion and uniform in distribution. Wang et al. employed a one-step surfactant-assisted hydrothermal synthesis to prepare ultra-thin Mg(OH)_2_ nanoflakes with a thickness of only 3–8 nm. The core of this method lies in utilizing sodium dodecyl sulfate (SDS) simultaneously as both a structure-directing agent and a surface modifier, enabling the nanoflakes to form a long-term stable (>2 months) colloidal suspension in water. This ensures molecular-level uniform distribution on paper fibers. Such excellent dispersibility directly translates into long-term protective performance: treated aged bamboo paper maintained a pH of 5.47 even after accelerated aging at 105 °C for five months, demonstrating significantly superior long-term anti-acidification capability compared to traditional particulate agents. Furthermore, this material also imparts flame retardancy to the paper, increasing the limiting oxygen index from 16% to 23%, thereby achieving multifunctional protection with a single material (Figure 5A) [51]. This provides an optimized solution for uniform, long-lasting deacidification and functional enhancement in the conservation of paper-based cultural relics.

### 4.2. Nano-Mg(OH)_2_ for Paper Deacidification and Protection

Meng et al. developed a GG-C-M nanocomposite hydrogel system based on nano-Mg(OH)_2_ (a gellan gum hydrogel loaded with sodium carboxymethyl cellulose-stabilized nano-Mg(OH)_2_ dispersion) (Figure 5B). Applied to a severely acidified newspaper from 1931 (Republic of China era) from the Special Collections of Liaoning University, as well as simulated acidified, dry-heat-aged, and UV-aged Xuan paper samples, the system achieved a synergistic effect of deacidification and reinforcement. It successfully restored severely acidified paper (initial pH ≤ 5.0) to a slightly alkaline state (pH 7.25–7.32), establishing a stable alkaline reserve of 0.27–0.34 mol/kg and significantly improving tensile strength (35.50–41.40 N). The treated paper demonstrated effective resistance to re-acidification. Examination revealed no particle residue on paper fibers, avoiding side effects commonly associated with traditional methods, such as edge curling or increased thickness. The system also offers advantages of biocompatibility and targeted local treatment, providing a mild, efficient, and durable conservation solution for fragile paper-based cultural relics [46].

### 4.3. Nanocomposite Treatment for Acidic Paper

To address issues associated with traditional single deacidifying agents—such as uncontrollable alkaline strength, insufficient alkaline reserve, non-uniform deacidification, and localized high alkalinity—Gui et al. developed an MgO/CaCO_3_ nanocomposite (featuring a near-hexagonal hollow/porous structure with a particle size of approximately 40 nm). By modulating the molar ratio of MgO to CaCO_3_ (3:1, 4:1, 5:1), controllable alkaline strength was achieved. Applied to acidic paper samples (initial pH 4.42), this material demonstrated significant deacidification efficacy. It combines the advantages of rapid and efficient deacidification from MgO with the long-lasting, mild deacidification provided by CaCO_3_. Post-treatment, the paper pH stabilized within a weakly alkaline range of 9.03–9.35, with an alkaline reserve of 0.285–0.301 mol/kg. Excellent deacidification uniformity was observed (ΔpH 0.31–0.36). Furthermore, the treatment caused no damage to the paper’s original optical or mechanical properties: the maximum decrease in ISO brightness was only 3.6%, and changes in tensile strength and tear resistance were minimal. Compared to a physical mixture of MgO and CaCO_3_, this nanocomposite demonstrated superior deacidification performance and enhanced uniformity, offering a flexible and controllable solution for preserving paper-based materials with varying degrees of acidification [40].

Building upon the foundation of precise deacidification, researchers have further pursued the integration of multiple functionalities into materials, combining deacidification with other preservation needs such as strengthening, UV resistance, and antimicrobial protection to develop multifunctional nanocomposite restoration materials. Li et al. developed a CMC-Ca(OH)_2_-TiO_2_ nanocomposite, employing carboxymethyl cellulose (CMC) as a carrier for the in situ growth of spindle-shaped Ca(OH)_2_ nanoparticles (approx. 30 nm) and the loading of TiO_2_ nanoparticles (approx. 5 nm). Applied to paper cultural relics containing carbon ink, cinnabar, carmine, and gamboge pigments, as well as Xuan paper samples, this material achieved integrated multifunctional restoration encompassing strengthening, deacidification, UV resistance, and antimicrobial effects. The composite exhibited over 85% transparency in the visible light range, ensuring no interference with the legibility of text or images. It successfully elevated the pH of acidic paper to a weakly alkaline level of 8.21, which remained above 7.88 even after dry-heat and damp-heat accelerated aging. Concurrently, it increased the paper’s tensile strength in the machine and cross directions by 24.6% and 17.4%, respectively. The strength loss after aging was only 20–30%, significantly lower than the 76–82% loss observed in untreated samples. The TiO_2_ component effectively absorbed ultraviolet light, reducing fiber degradation and resulting in a color difference ΔE < 2 for pigments, thus preventing noticeable fading or discoloration. Additionally, it formed inhibition zones with diameters of 2.36–5.38 mm against molds such as Aspergillus flavus and Aspergillus niger. This approach significantly enhances the long-term preservation stability of paper-based cultural relics and streamlines the traditionally multi-step restoration process [50].

The core advantage of nano-deacidification technology lies in its exceptional permeability and uniformity, enabling “inside-out” deep protection. This makes it particularly suitable for precious artifacts with high aesthetic requirements or those with severely embrittled fibers that require profound reinforcement [3,15,61,69]. In contrast, traditional deacidification methods remain irreplaceable for large-scale, batch processing of ordinary acidic documents and archives, owing to their technological maturity and controllable costs [70,71].

## 5. Challenges, Limitations, and Future Prospects

Despite its considerable potential, the transition of nano-deacidification technology from the laboratory to widespread conservation practice faces a series of challenges.

### 5.1. Key Current Challenges and Limitations

#### 5.1.1. Lack of Long-Term Stability and Durability Data

Most studies report on initial treatment effects or outcomes following only a few months of accelerated aging. The chemical stability of nanomaterials and their reaction products over the timescales relevant to actual conservation environments (decades to centuries), the long-term evolution of their interface with cellulose fibers, and their complex influence on paper aging mechanisms require more extended longitudinal studies and data accumulation [4,27,28].

#### 5.1.2. Absence of a Standardized Evaluation System

Currently, research teams employ diverse testing methods, aging conditions, and efficacy metrics, making direct and accurate comparison between different studies difficult. There is a pressing need to establish industry or national standards specifically for nano-deacidification technology to regulate the entire process, from material characterization to efficacy assessment [32,36]. A robust standard should include: (1) Material characterization protocols mandating the reporting of nanoparticle size distribution (via dynamic light scattering or TEM), crystallinity (XRD), surface area, and zeta potential in the intended dispersion medium; (2) Standardized accelerated aging protocols specifying conditions (e.g., dry heat at 80 °C or 105 °C, damp heat, light exposure) and durations relevant to predicting long-term behavior; (3) Minimum performance thresholds, such as a target pH range (e.g., 7.5–8.5), a minimum alkaline reserve (e.g., >0.2 mol/kg as CaCO_3_ equivalent), and maximum allowable changes in mechanical strength (e.g., ΔTS < ±15%) and color (ΔE* < 3) post-treatment and after accelerated aging; and (4) Reporting requirements for application parameters (concentration, method, drying conditions) to ensure reproducibility. The development of such a consensus framework is critical for validating treatment efficacy, facilitating technology transfer, and building trust within the conservation community.

#### 5.1.3. Scalable Production and Application Costs

The synthesis and purification of nanomaterials at the laboratory scale remain costly. Scaling up to industrial production levels capable of meeting the demands of large-scale treatment, while ensuring consistent product quality and stability, constitutes a key bottleneck for commercialization. Furthermore, the optimization of application processes (e.g., automated spraying, drying control) requires parallel development [41]. Critically, a comprehensive cost-effectiveness analysis comparing nano-treatments to conventional methods is largely absent. Traditional mass deacidification (e.g., using micrometer-sized alkaline particles) benefits from economies of scale and mature infrastructure, resulting in a significantly lower cost per item and is thus suitable for large-scale archival processing [70,71]. In contrast, current nano-deacidification processes, especially those involving specialized solvents or composite materials, incur higher costs per treatment. These costs stem from expensive nanomaterial synthesis, potential solvent recovery needs, and the often slower, more meticulous application procedures required for individual artifacts. Therefore, from an economic perspective, nano-deacidification is currently positioned as a high-value, precision tool for selected high-priority items rather than a direct, cost-effective replacement for all traditional methods.

#### 5.1.4. Safety and Ethical Considerations for Complex Heritage Substrates

The application of nanomaterials to actual heritage objects, which often possess complex and heterogeneous compositions (e.g., various pigments, inks, adhesives, coatings), necessitates rigorous safety and compatibility assessments. The high reactivity of nanoparticles requires systematic investigation to preclude unforeseen chemical interactions with non-cellulosic components, such as discoloration induced in sensitive pigments or inks. Although promising studies on specific media like iron gall ink exist [21,55], comprehensive databases on the interactions between a wide range of nanomaterials and diverse historical materials remain lacking, making pre-treatment testing on representative samples an essential yet often underreported practice. Furthermore, this intervention at the nanoscale prompts critical ethical reflection, particularly regarding its alignment with core conservation principles such as “minimal intervention” and “re-treatability.” The strong, often intimate binding of nanoparticles to cellulose fibers renders the treatment technically almost irreversible, challenging the traditional preference for physically reversible treatments and necessitating a careful re-examination of conservation ethics within the context of advanced nanotechnology.

#### 5.1.5. Environmental, Health, and Regulatory Considerations of Solvent Systems

A significant practical challenge concerns the dispersion media, particularly fluorocarbon solvents, which are favored in many studies for their inertness and good dispersion properties. However, their use raises important environmental and health concerns. Many fluorocarbons are persistent in the environment, have a high global warming potential, and their handling poses risks of inhalation exposure to operators, requiring strict ventilation controls and personal protective equipment [12,32,36]. Furthermore, the use of such solvents must navigate evolving regulatory frameworks governing volatile organic compounds (VOCs), per- and polyfluoroalkyl substances (PFAS), and greenhouse gases. This regulatory landscape complicates their widespread adoption in conservation practice and underscores the urgent need for research into greener, safer, and compliant alternative dispersion systems (e.g., modified alcohols, certain siloxanes, or aqueous-based systems with improved stability).

### 5.2. Outlook for Future Research Directions

#### 5.2.1. Development of Intelligent and Green Nanomaterials

(1) Stimuli-Responsive Materials: Research into “intelligent” nano-carriers capable of responding to localized pH changes in paper and actively releasing alkaline agents. (2) Multifunctional Composite Materials: Develop “all-in-one” nanomaterials integrating deacidification, antimicrobial, and strengthening functionalities to minimize the stress of repeated treatments on cultural artifacts. (3) Bio-Based and Green Materials: Explore the use of bio-based materials, such as cellulose nanocrystals, as carriers or reinforcing phases to develop fully green and sustainable deacidification systems. (4) Green Solvent and Dispersion Technology: A critical research frontier is the development of high-performance, stable nano-dispersions in environmentally benign and low-toxicity solvents. This includes optimizing systems in short-chain alcohols, exploring novel green solvents (e.g., certain terpenes, biosourced esters), or advancing aqueous colloidal science to achieve long-term stability without compromising penetration or efficacy.

#### 5.2.2. Deepening Fundamental Research and Standardization Efforts

Utilize advanced characterization techniques (e.g., in situ SEM, micro-XRD, Atomic Force Microscopy) to gain deeper insights into the migration of nanoparticles within paper, their reaction kinetics, and the micro-mechanics at interfaces. Promote interdisciplinary collaboration to establish universally accepted accelerated aging protocols and long-term performance databases, and to formulate technical standards and operational guidelines. A specific and urgent need is the development of standardized testing protocols and the creation of reference databases to systematically evaluate the compatibility of nano-deacidification agents with a comprehensive range of historical media (pigments, inks, adhesives). This will enable informed, risk-based decision-making for treatment planning.

#### 5.2.3. Promoting Technological Transfer and Engineering Applications

Optimize technologies for the large-scale, low-cost synthesis and stabilization of nanomaterials. Develop dedicated application equipment and process workflows suitable for different types of artifacts and binding structures. Conduct more pilot-scale projects and trials on actual cultural heritage objects to accumulate practical experience and assess the overall cost-effectiveness. Future work must include rigorous techno-economic analyses (TEA) and life-cycle assessments (LCA) to transparently compare the full economic and environmental costs of nano-treatments against conventional and emerging alternatives. This will clarify the niche for nano-deacidification within the broader conservation toolkit and guide resource allocation.

#### 5.2.4. Strengthening Interdisciplinary Dialogue and Ethical Framework Development

Establish regular interdisciplinary dialogue mechanisms among materials scientists, chemists, conservators, art historians, and ethicists. Collaboratively develop ethical guidelines and application principles for cultural heritage conservation that adapt to the advancement of new technologies.

## 6. Conclusions

Nanomaterials, particularly nano-calcium hydroxide and nano-magnesium hydroxide, offer a highly promising innovative solution for the deacidification and preservation of paper-based cultural heritage. Their nanoscale characteristics enable deep, uniform penetration, coupled with efficient acid neutralization and long-term buffering capacity, thereby effectively addressing the limitations of conventional deacidification techniques in terms of microscopic uniformity and depth of treatment. Through systematic research into material preparation, dispersion stabilization, application methodologies, and multi-dimensional performance evaluation, this technology has progressed from a laboratory concept to the verge of practical application.

However, its true maturation and widespread adoption depend on a deeper understanding of the long-term durability of the materials, the establishment of standardized evaluation protocols, the reduction in costs for large-scale implementation, and careful consideration of safety and conservation ethics concerning complex cultural heritage systems. Future research should focus on developing more intelligent and environmentally friendly multifunctional nano-systems, while fostering closer integration between fundamental science, engineering technology, and conservation practice.

It is foreseeable that nano-deacidification technology will not entirely replace traditional methods but will instead become a sophisticated, high-end option in the conservator’s toolkit. It is particularly suited for highly valuable, fragile paper artifacts with stringent requirements regarding treatment aesthetics. As the associated challenges are gradually overcome, nanotechnology holds the potential to guide paper conservation into a new era characterized by greater precision, efficiency, and sustainability.

## Figures and Tables

**Table 1:** Comparative overview of nano-Ca(OH)_2_ and nano-Mg(OH)_2_ as deacidification agents.

Aspect	Nano-Ca(OH)_2_	Nano-Mg(OH)_2_	Refs.
Alkalinity	Strong (pH~12.4)	Moderate (pH~10.5)	[4,28,36,41]
Long-term buffer form	CaCO_3_ (high chemical stability)	MgCO_3_ (may convert to basic carbonates under humidity)	[28,30,35,41]
Risk to sensitive pigments	Higher, due to strong alkalinity; may alter pH-sensitive dyes/inks	Lower, milder alkalinity reduces risk of pigment alteration	[21,28,30,32]
Suitability for paper types	Preferred for highly acidic, robust papers; may risk embrittlement if over-applied	Preferred for historically valuable papers or those with sensitive media	[30,39,41]
Dispersion stability	Lower; prone to carbonation in air, requires sealed/low-temperature storage	Generally better; less reactive toward atmospheric CO_2_	[4,28,39,41]
Cost & availability	Lower cost, widely available precursors	Slightly higher cost, but still economically feasible	[4,32,41]

**Table 2:** Core Assessment Metrics and Methodologies for the Efficacy of Nano-Deacidification Treatment.

Dimension	Metric	Key Methods	Significance/Criteria
Acid Neutralization	Surface pH	Cold Extraction (TAPPI T509), Surface Electrode [28,57,58]	Target pH: 7–8.5 (neutral to slightly alkaline).
Alkaline Reserve	Carbonate Content (as CaCO_3_)	Titration, XRF [59,60,61]	Quantifies long-term buffering capacity against future acidification.
Physical Properties	Tensile Strength, Folding Endurance, Tear Resistance	Universal Testing Machine (ISO standards) [57,60]	Treatment should not degrade, and may slightly enhance, mechanical properties.
Optical Properties	Color Change (ΔE*)	Chroma Meter (CIE L*a*b*) [60,62,63]	ΔE* < 3 is typically acceptable; minimal visible discoloration.
Morphology & Distribution	Particle Distribution & Penetration Depth	SEM-EDS [57,60]	Visualizes nanoparticle adhesion on fibers and cross-sectional distribution.
Aging Stability	Performance Change after Accelerated Aging	Dry/Heat, Damp/Heat, or Light Aging [46,57,64]	Evaluates treatment durability; key properties (pH, strength) should remain stable.

## Data Availability

No new data were created or analyzed in this study.
